# Supplementary material for: Over-representation of potential SP4 target genes within schizophrenia-risk genes
Source: Mol Psychiatry. 2021 Nov 8;27(2):849–54. doi: 10.1038/s41380-021-01376-8 (PMC9054665; doi:10.1038/s41380-021-01376-8)
Supplement: Supplementary file 1 — Supplemental Figure Legend [file 41380_2021_1376_MOESM1_ESM.docx]

**Supplemental Figure Legend**

**Supplemental Figure S1. The GC-box containing genes have a higher GC content in their proximal promoter regions.** The genes containing at least one GC-box (Y) and the genes without the GC-box (N) were separated for boxplotting their GC contents in different groups of genes. The GC-box containing genes have a significantly higher GC content (F(1,17282)=7739.34, p < 2 x 10^-16^) than genes without GC-box across all groups regardless of diseases. SCZ_S1 (SCHEMA1, risk-genes with p < 10^-4^), SCZ_S2 (SCHEMA2, risk-genes with p < 10^-3^), SCZ_G1 (GWAS1, FINEMAP prioritized genes), SCZ_G2 (GWAS2, all prioritized genes).
