# Supplementary figures and images for: Over-representation of potential SP4 target genes within schizophrenia-risk genes
Source: Mol Psychiatry. 2021 Nov 8;27(2):849–54. doi: 10.1038/s41380-021-01376-8 (PMC9054665; doi:10.1038/s41380-021-01376-8)

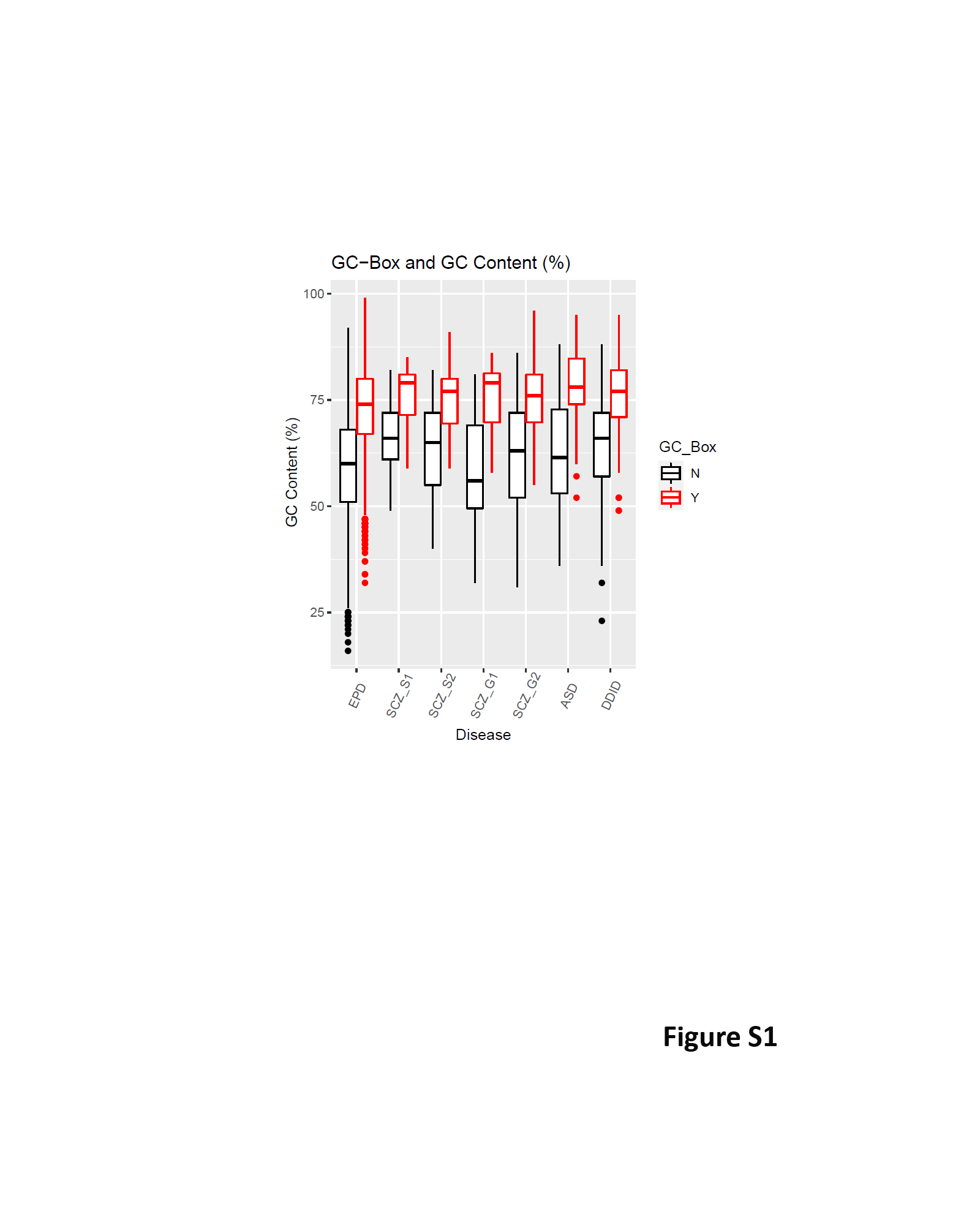

Supplement: Supplementary file 2 — Supplemental Figure S1 [file 41380_2021_1376_MOESM2_ESM.tif]
