# Supplementary material for: Over-representation of potential SP4 target genes within schizophrenia-risk genes
Source: Mol Psychiatry. 2021 Nov 8;27(2):849–54. doi: 10.1038/s41380-021-01376-8 (PMC9054665; doi:10.1038/s41380-021-01376-8)
Supplement: Supplementary file 6 — Supplemental Table S4 [file 41380_2021_1376_MOESM6_ESM.docx]

Permutation tests (N=9,999) for the GC box numbers

Disease p_value

1 SCZ_SCHEMA1 0.6167617

2 SCZ_SCHEMA2 0.8805881

3 SCZ_GWAS1 0.9399940

4 SCZ_GWAS2 0.2154215

5 ASD 0.1905191

6 DDID 0.4580458

|  |  | |  |  |  |  |  |  |
| --- | --- | --- | --- | --- | --- | --- | --- | --- |
| **Welch's t-tests for the GC-content and FDR adjusted p values** | | | | | | | |  |
|  | | **SCZ_S1** | **SCZ_S2** | **SCZ_G1** | **SCZ_G2** | **ASD** | **DD/ID** |  |
| **mean of EPD** | | 0.6563 | 0.6563 | 0.6563 | 0.6563 | 0.6563 | 0.6563 |  |
| **mean of risk genes** | | 0.7254 | 0.7077 | 0.6771 | 0.6840 | 0.6959 | 0.7059 |  |
| **ci.lower** | | -0.1068 | -0.0830 | -0.0569 | -0.0416 | -0.0682 | -0.0643 |  |
| **ci.upper** | | -0.0312 | -0.0196 | 0.0153 | -0.0138 | -0.0109 | -0.0348 |  |
| **p.value** | | **0.0009** | **0.0021** | 0.2533 | **0.0001** | **0.0073** | **1.98E-10** |  |
| **p_FDR** | | **0.0017** | **0.0031** | 0.2533 | **0.0003** | **0.0088** | **1.19E-09** |  |
|  |  | |  |  |  |  |  |  |
